# Hyperspectral Imaging—A Novel Tool to Assess Tissue Perfusion and Oxygenation in Esophageal Anastomoses

**DOI:** 10.1055/s-0043-1769106

**Published:** 2023-06-12

**Authors:** Duarte Vaz Pimentel, Larissa Merten, Jan-Hendrik Gosemann, Ines Gockel, Boris Jansen-Winkeln, Steffi Mayer, Martin Lacher

**Affiliations:** 1 Department of Pediatric Surgery, University Hospital Leipzig, Leipzig, Sachsen, Germany; 2 Department of Visceral, Transplantation, Thoracic and Vascular Surgery, University Hospital Leipzig, Leipzig, Sachsen, Germany

**Keywords:** perfusion, hyperspectral imaging, esophageal atresia, anastomotic stricture, anastomotic leakage

## Abstract

Anastomotic stricture and leakage are common complications after repair of esophageal atresia (EA). A compromised perfusion of the anastomosis is a contributing factor. Hyperspectral imaging (HSI) is an ultrashort noninvasive method to measure tissue perfusion. We present two cases of with tracheoesophageal fistula (TEF)/EA repair, in whom we applied HSI: the first patient was a newborn with EA type C who underwent open TEF repair. The second one had an EA type A and cervical esophagostomy, in whom we performed gastric transposition. In both patients, HSI confirmed a good tissue perfusion of the later anastomosis. The postoperative course was uneventful and both patients are on full enteral feeds. We conclude that HSI is a safe and noninvasive tool that allows near real-time assessment of tissue perfusion and can contribute to the identification of the optimal anastomotic region during pediatric esophageal surgery.

## Introduction

Esophageal atresia (EA) with or without tracheoesophageal fistula (TEF) has an incidence of 1 in 4,099 births. 1 Anastomotic stricture and leakage are frequent problems following repair of EA/TEF, often due to tension on or compromised perfusion of the anastomosis. 2 3 Anastomotic strictures require 5.1 ± 5.6 dilatations on average. Almost all patients require at least one readmission within the first year of life. 4 The intraoperative assessment of tissue perfusion has recently gained increasing interest in gastrointestinal surgery. 5 Different methods have been established, the most prevalent being indocyanine green fluorescence scan. 6 7 A drawback of this and other techniques to assess tissue perfusion is the need to inject contrast agents with potential adverse reactions and toxicity. 8

Hyperspectral imaging (HSI) is a noninvasive tool for the assessment of tissue perfusion and oxygenation based on the tissue-specific reflection of light in hyperspectral ranges of visible and near-infrared light (500–1,000 nm wavelength). 9 It does not require contrast agents and provides near real-time information on tissue perfusion (near-infrared perfusion index [NIR-PI]), tissue hemoglobin index (THI), and tissue oxygen saturation (StO _2_ ) with 10 to 15 seconds delay.

HSI has been applied to various fields of surgery including cutaneous flaps in reconstructive surgery, partial nephrectomies, or intestinal anastomoses. 10 11 12 13 We report the first application of HSI in pediatric esophageal surgery.

## Case Description

**Fig. 1:**
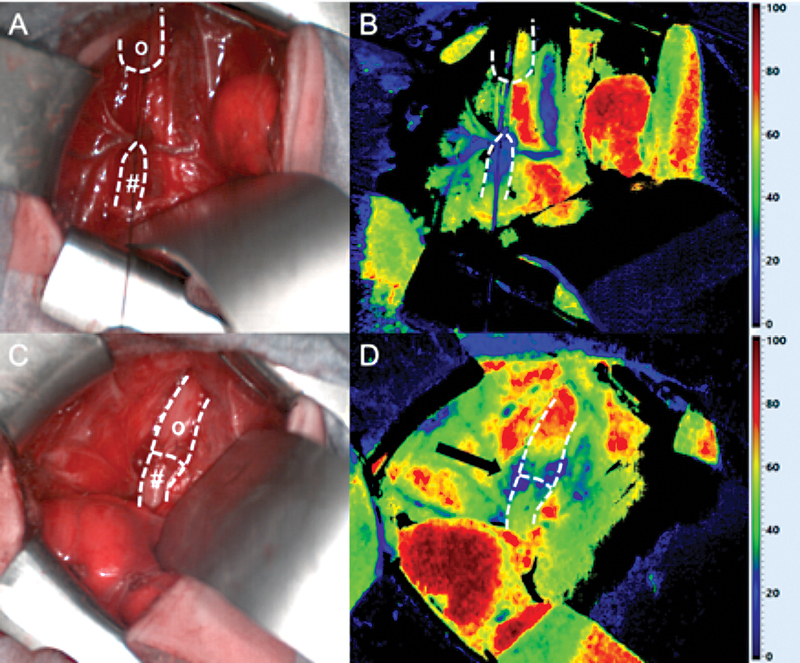
Intraoperative situs ( **A, C** ) and HSI StO _2_ ( **B, D** ) after TEF ligation: Well-perfused upper esophageal pouch ( *o* ) and impaired perfusion of the lower esophagus ( *#* ) before resection of its distal tip ( **A, B** ). After resection of the tip of the distal pouch (Fig. 2), improved perfusion with only minor impairment at the anastomotic suture line itself ( *arrow* ) was detected ( **C, D** ). HSI, hyperspectral imaging; StO _2_ , tissue oxygen saturation; TEF, tracheoesophageal fistula.

**Fig. 2:**
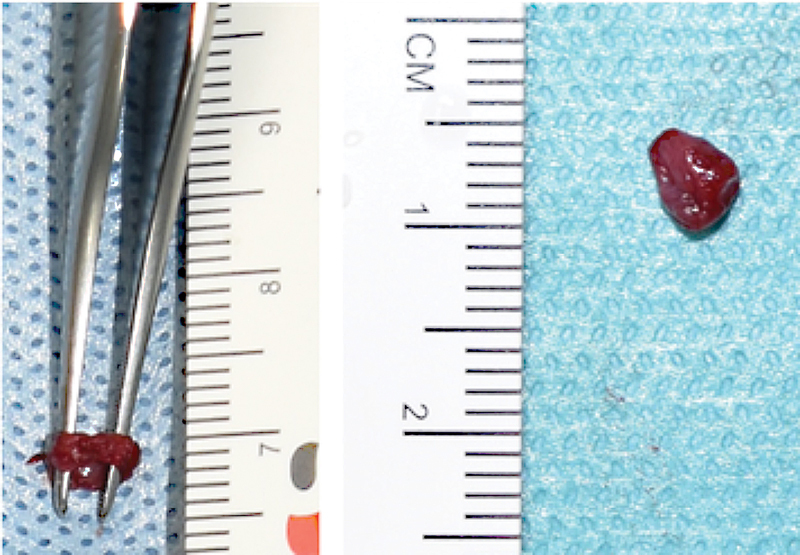
Additional resection of 2 mm distal esophagus after hyperspectral imaging measurement.

*Case 1* : A newborn girl (body weight 1,850 g, gestational age 36 weeks) presented with VACTERL association including EA type C, anorectal malformation with vestibular fistula, and septum defect. She underwent open TEF repair on day 2 of life. After TEF closure, the tip of the lower esophagus showed an impaired perfusion on real-time HSI assessment (NIR-PI 58%; THI 100%; StO _2_ 40%; Fig. 1A, B ). Thus, an additional 2 mm of the lower esophagus ( Fig. 2 ) were resected to accomplish an anastomosis in a well-perfused area (NIR-PI 92%; THI 100%; StO _2_ 55%; Fig. 1C, D ). The postoperative course was uneventful. No anastomotic leak or stenosis occurred. The patient was discharged home on postoperative day 41 due to difficulties related to her low birth weight and cardiac situation. During the further course of 22 months, no esophageal dilatations were required.

**Fig. 3:**
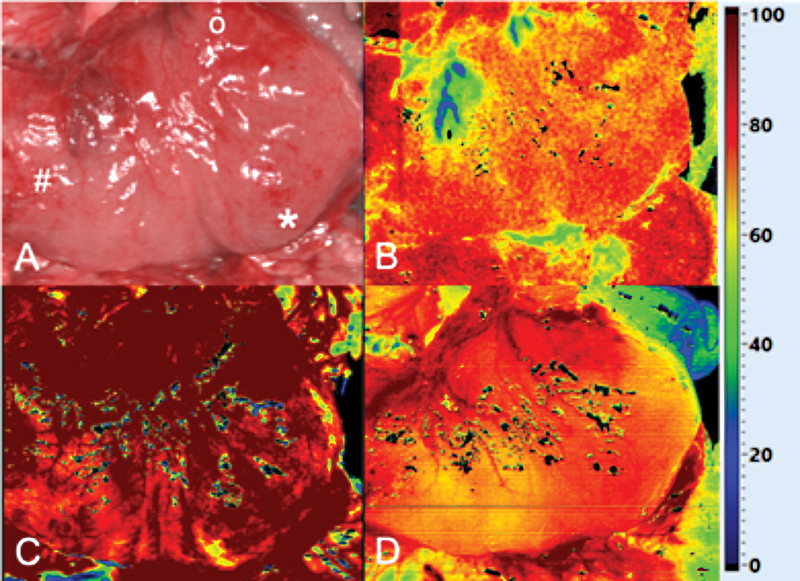
Intraoperative situs ( **A** ) and HSI assessment (StO _2_ [ **B** ], THI [ **C** ], and NIR-PI [ **D** ]) before esophagogastric anastomosis for gastric transposition. *o, fundus* ; *, *greater curvature;* #, *antrum* ; HSI, hyperspectral imaging; NIR-PI, near-infrared perfusion index; StO _2_ , tissue oxygen saturation; THI, tissue hemoglobin index.

*Case 2* : A 9-month-old boy with EA type A underwent gastric transposition after receiving a cervical esophagostomy in the newborn period at another institution. After laparotomy, a good perfusion of the gastric fundus was confirmed and a cervical esophagogastric anastomosis was established (StO _2_ 80%, THI 100%, NIR-PI 80%; Fig. 3 ). The subsequent course remained uneventful for 26 months. No dilation of the cervical anastomosis was required. The patient is on full oral feeds.

## Discussion

Anastomotic leakage and stricture after repair of EA/TEF occur in ∼20 and 28% of cases, respectively. 14 This is, among other factors, caused by an impaired perfusion of the anastomosis. 15 Although intraoperatively the perfusion of the lower esophagus appeared unaltered macroscopically in patient 1, HSI measurement revealed a diminished perfusion of the distal pouch. Thus, HSI had an additional diagnostic value. We therefore resected additional 2 mm of the distal esophagus to create an anastomosis of the two esophageal ends, which were well perfused. Of note, we recognized an impaired perfusion at the anastomosis itself, most likely from the suture line ( Fig. 1 ), which has been described in adults before. 11

Currently, several clinical studies are ongoing to evaluate the benefits of HSI in gastrointestinal anastomoses. A more peripheral adaptation of the anastomosis based on intraoperative HSI was reported for oncologic esophagectomy in 38% of patients without any postoperative leak. 16 In another study reporting on colorectal resection, a deviation between the transection line planned by the surgeon and the border line visualized by HSI of 1 to 13 mm was found for all patients. Consequently, the resection area was corrected proximally in 21% of patients due to the intraoperative HSI records. Thus, the authors concluded that the determination of the resection margin by HSI provide the surgeon with an objective decision aid for assessment of the best possible perfusion and ideal anastomotic area in colorectal surgery. 17 Its transferability to other gastrointestinal anastomoses is obvious. However, normal or cutoff values for different gastrointestinal anastomotic types especially in pediatric surgery are still required.

Besides HSI, other methods such as indocyanine green fluorescence can also assess intraoperative perfusion and guide surgeons to improve surgical outcomes. 18 However, this technique requires contrast agents, which is not the case in HSI. HSI is a safe, fast, noninvasive technique that can easily be implemented during surgery. The tissue perfusion assessed by HSI is also comparable to that of indocyanine green fluorescence, which has recently been shown for colorectal resections as well as oncologic esophagectomy. 16 19 HSI has also been shown to discriminate tissue perfusion in acute mesenteric ischemia and depict tissue viability via reflectance spectra. 20 Besides, it has been applied to evaluate liver and gastric perfusion during pancreatoduodenectomy and identifying exact resection planes for anatomic liver resection. 21 22

Only recently, also, a HSI system for minimally invasive surgery has been introduced. The HSI laparoscope available has a diameter of 10 mm and is consistent for object distances up to 10 cm. 23 24 It is currently examined for clinical practicability and impact in adult surgery.

These emerging experiences in adult surgery indicate promising applications in pediatric surgery. Future tasks include the implementation of HSI measurements in surgical interventions where adequate tissue perfusion plays a key role. However, to date, there are no reliable normal and cutoff measurements for decision-making. Moreover, prospective studies comparing intraoperative perfusion with postoperative outcomes are also lacking. However, we think that the HSI technique is easy to perform and a promising tool to assess perfusion in pediatric surgery, which may contribute to better in children.

## Conclusion

This is the first report on HSI assessment in pediatric surgery as a safe and noninvasive tool to assess tissue perfusion in real time. It can help determine the optimal anastomotic region during pediatric esophageal surgery.

## Notes

New Insights and the Importance for a Pediatric Surgeon We describe the first use of HSI (a safe and noninvasive tool that allows near real-time assessment of tissue perfusion and can contribute to the identification of the optimal anastomotic region) in pediatric esophageal surgery.

## References

[1] Nassar N Leoncini E Amar E Prevalence of esophageal atresia among 18 international birth defects surveillance programs Birth Defects Res A Clin Mol Teratol 2012 94 11 893 899 22945024 10.1002/bdra.23067 PMC4467200

[2] Askarpour S Peyvasteh M Javaherizadeh H Askari N Evaluation of risk factors affecting anastomotic leakage after repair of esophageal atresia Arq Bras Cir Dig 2015 28 03 161 162 26537137 10.1590/S0102-67202015000300003 PMC4737352

[3] Touloukian R J Seashore J H Thirty-five-year institutional experience with end-to-side repair for esophageal atresia Arch Surg 2004 139 04 371 374, discussion 374 15078702 10.1001/archsurg.139.4.371

[4] Dingemann C Dietrich J Zeidler J Early complications after esophageal atresia repair: analysis of a German health insurance database covering a population of 8 million Dis Esophagus 2016 29 07 780 786 25893931 10.1111/dote.12369

[5] Urbanavičius L Pattyn P de Putte D V Venskutonis D How to assess intestinal viability during surgery: a review of techniques World J Gastrointest Surg 2011 3 05 59 69 21666808 10.4240/wjgs.v3.i5.59 PMC3110878

[6] Alander J T Kaartinen I Laakso A A review of indocyanine green fluorescent imaging in surgery Int J Biomed Imaging 2012 2012 940585 22577366 10.1155/2012/940585 PMC3346977

[7] Turner S R Molena D R The role of intraoperative fluorescence imaging during esophagectomy Thorac Surg Clin 2018 28 04 567 571 30268302 10.1016/j.thorsurg.2018.07.009 PMC6166438

[8] Alford R Simpson H M Duberman J Toxicity of organic fluorophores used in molecular imaging: literature review Mol Imaging 2009 8 06 341 354 20003892

[9] Lu G Fei B Medical hyperspectral imaging: a review J Biomed Opt 2014 19 01 10901 24441941 10.1117/1.JBO.19.1.010901 PMC3895860

[10] Akbari H Kosugi Y Kojima K Tanaka N Hyperspectral imaging and diagnosis of intestinal ischemia Annu Int Conf IEEE Eng Med Biol Soc 2008 2008 1238 1241 19162890 10.1109/IEMBS.2008.4649387

[11] Jansen-Winkeln B Maktabi M Takoh J P Hyperspektral-Imaging bei gastrointestinalen Anastomosen Chirurg 2018 89 09 717 725 29637244 10.1007/s00104-018-0633-2

[12] Best S L Thapa A Jackson N Renal oxygenation measurement during partial nephrectomy using hyperspectral imaging may predict acute postoperative renal function J Endourol 2013 27 08 1037 1040 23544949 10.1089/end.2012.0683

[13] Gockel I Jansen-Winkeln B Holfert N Möglichkeiten und Perspektiven der Hyperspektralbildgebung in der Viszeralchirurgie Chirurg 2020 91 02 150 159 31435721 10.1007/s00104-019-01016-6

[14] Du J Huang J Li Y Chen Y Guo W Hou D The repair of esophageal atresia and major complications—a systematic review and our experience in dealing with the tracheoesophageal fistula Ann Laparosc Endosc Surg 2019 4 90 90

[15] Mayer S Gitter H Göbel P Behandlung der Ösophagusatresie mit unterer tracheoösophagealer Fistel – Zusammenfassung der aktuellen S2K-Leitlinie der DGKCH Klin Padiatr 2020 232 04 178 186 32590849 10.1055/a-1149-9483

[16] Hennig S Jansen-Winkeln B Köhler H Novel intraoperative imaging of gastric tube perfusion during oncologic esophagectomy-a pilot study comparing hyperspectral imaging (HSI) and fluorescence imaging (FI) with indocyanine green (ICG) Cancers (Basel) 2021 14 01 97 35008261 10.3390/cancers14010097 PMC8750976

[17] Jansen-Winkeln B Holfert N Köhler H Determination of the transection margin during colorectal resection with hyperspectral imaging (HSI) Int J Colorectal Dis 2019 34 04 731 739 30712079 10.1007/s00384-019-03250-0

[18] Lau C T Au D M Wong K KY Application of indocyanine green in pediatric surgery Pediatr Surg Int 2019 35 10 1035 1041 31243546 10.1007/s00383-019-04502-4

[19] Jansen-Winkeln B Germann I Köhler H Comparison of hyperspectral imaging and fluorescence angiography for the determination of the transection margin in colorectal resections-a comparative study Int J Colorectal Dis 2021 36 02 283 291 32968892 10.1007/s00384-020-03755-z PMC7801293

[20] Mehdorn M Köhler H Rabe S M Hyperspectral imaging (HSI) in acute mesenteric ischemia to detect intestinal perfusion deficits J Surg Res 2020 254 7 15 32402834 10.1016/j.jss.2020.04.001

[21] Moulla Y Buchloh D C Köhler H Hyperspectral imaging (HSI)-a new tool to estimate the perfusion of upper abdominal organs during pancreatoduodenectomy Cancers (Basel) 2021 13 11 2846 34200412 10.3390/cancers13112846 PMC8201356

[22] Sucher R Athanasios A Köhler H Hyperspectral imaging (HSI) in anatomic left liver resection Int J Surg Case Rep 2019 62 108 111 31493663 10.1016/j.ijscr.2019.08.025 PMC6731347

[23] Pfahl A Köhler H Thomaßen M T Video: clinical evaluation of a laparoscopic hyperspectral imaging system Surg Endosc 2022 36 10 7794 7799 35546207 10.1007/s00464-022-09282-y PMC9485189

[24] Köhler H Kulcke A Maktabi M Laparoscopic system for simultaneous high-resolution video and rapid hyperspectral imaging in the visible and near-infrared spectral range J Biomed Opt 2020 25 08 86004 10.1117/1.JBO.25.8.086004 PMC7453262 32860357

